# CGLCS-Net: Addressing Multi-Temporal and Multi-Angle Challenges in Remote Sensing Change Detection

**DOI:** 10.3390/s25092836

**Published:** 2025-04-30

**Authors:** Ke Liu, Hang Xue, Caiyi Huang, Jiaqi Huo, Guoxuan Chen

**Affiliations:** 1 North China Institute of Aerospace Engineering, College of Remote Sensing and Information Engineering, Langfang 065000, China; liuke1176@nciae.edu.cn (K.L.); huangcy@stumail.nciae.edu.cn (C.H.); huojiaqi@stumail.nciae.edu.cn (J.H.); chenguoxuan@stumail.nciae.edu.cn (G.C.); 2 Hebei Collaborative Innovation Centre of Aerospace Remote Sensing Information Processing and Application, Langfang 065000, China

**Keywords:** high-resolution remote sensing (RS) image, change detection, deep learning, Global-Local Context

## Abstract

Currently, deep learning networks based on architectures such as CNN and Transformer have achieved significant advances in remote sensing image change detection, effectively addressing the issue of false changes due to spectral and radiometric discrepancies. However, when handling remote sensing image data from multiple sensors, different viewing angles, and extended periods, these models show limitations in modelling dynamic interactions and feature representations in change regions, restricting their ability to model the integrity and precision of irregular change areas. We propose the Context-Aware Global-Local Subspace Attention Change Detection Network (CGLCS-Net) to resolve these issues and introduce the Global-Local Context-Aware Selector (GLCAS) and the Subspace-based Self-Attention Fusion (SSAF) module. GLCAS dynamically selects receptive fields at different feature extraction stages through a joint pooling attention mechanism and depthwise separable convolution, enhancing global context and local feature extraction capabilities and improving feature representation for multi-scale and irregular change regions. The SSAF module establishes dynamic interactions between dual-temporal features via feature decomposition and self-attention mechanisms, focusing on semantic change areas to address challenges such as sensor viewpoint variations and the texture and spectral inconsistencies caused by long periods. Compared to ChangeFormer, CGLCS-Net achieved improvements in the IoU metric of 0.95%, 9.23%, and 13.16% on the three public datasets, i.e., LEVIR-CD, SYSU-CD, and S2Looking, respectively. Additionally, it reduced model parameters by 70.05%, floating-point operations by 7.5%, and inference time by 11.5%. These improvements enhance its applicability for continuous land use and land cover change monitoring.

## 1. Introduction

Change detection is a critical technology for identifying surface or scene changes by analyzing observation data from different periods. It is widely used in urban development [1,2], disaster management [3,4], deforestation [5,6], and environmental surveillance [7,8]. High-resolution remote sensing images can provide high-precision information about change regions. Still, two main challenges need to be addressed: On one hand, high-resolution images are known for their high spatial resolution and detailed information representation, which allows for the clear detection of object boundaries and subtle changes. However, this also introduces significant fine-grained noise, which imposes higher requirements on the robustness and sensitivity of detection models [9]. On the other hand, some remote sensing data are influenced by external factors such as weather and terrain due to the variety of platforms and sensor types, as well as different shooting angles and times during the imaging process [10]. This results in the same object displaying varying textures and spectral features across images, thereby increasing the complexity of feature alignment and model training [11]. Therefore, when processing multi-source, multi-temporal, and multi-angle high-resolution remote sensing images, the demands for change detection technology continue to rise. Models are required to have dynamic interaction modelling capabilities and accurate feature expression abilities.

Currently, commonly used change detection techniques are primarily based on CNN [12] and Transformer [13]. CNN-based networks for change detection typically perform initial feature extraction on dual-temporal images using an encoder with shared weights (such as ResNet [14], MobileNet [15], etc.) and apply differential methods [16,17], spatiotemporal attention mechanisms [18,19,20], or multi-scale feature fusion [21,22,23] to identify change regions. These methods achieve high precision and efficiency in capturing details and semantic information in change areas. For example, FC-Siam-conc [24] and FC-Siam-diff [24], based on a Siamese CNN architecture, fuse dual-temporal features through feature concatenation and feature differencing, respectively, to enhance the representation of change regions. DTCDSCN [25] uses a spatiotemporal attention mechanism to enhance the network’s focus on change regions, improving detection performance in key areas. SNUNet [26] addresses the loss of localization information in deep networks through dense connections, thus improving detection robustness. A2Net [27] integrates multi-scale features to effectively combine information from different scales, enhancing the network’s ability to extract change features. However, the local receptive field of CNNs is ineffective at expressing global context information and handling long-range dependencies, making it challenging to process scenarios that require global information [28]. Additionally, due to the fixed receptive field limitations in the same feature extraction layer [29], CNNs struggle to model irregular change regions, affecting the accuracy and detail preservation in change detection. In comparison, Transformer models can capture long-distance dependencies between features and excel at modelling global context information for complex scenes [30]. For instance, ChangeFormer [31] is a model built entirely on the Transformer architecture, using a decoder to generate difference maps from results computed by specific modules to complete the detection task. However, Transformers’ high dependency on large-scale labelled data and rapid computational complexity growth limit their use in high-resolution remote sensing image change detection [32].

Researchers have gradually explored hybrid architectures combining CNN and Transformer to address the abovementioned issues [33]. The TransUNetCD proposed by Li et al. [34] leverages the strengths of both CNN and Transformer, achieving complementarity by using CNN for local feature extraction and Transformer for global relationship modelling, resulting in an end-to-end encoder-decoder architecture for change detection. BIT, proposed by Chen et al. [35], enhances computational efficiency by combining CNN’s initial feature extraction ability with the Transformer’s non-local self-attention mechanism, significantly reducing the model’s resource demands in high-resolution image processing. The EATDer network, designed by Ma et al. [36], uses a Siamese architecture, with each branch capturing local and global information through three Self-Adaptive Vision Transformer (SAVT) blocks. An edge-aware decoder ensures clearer and smoother edges. DMINet, proposed by Feng et al. [37], integrates self-attention (SelfAtt) and cross-attention (CrossAtt), using a joint-time attention (JointAtt) block to regulate the global feature distribution of each input. This mechanism facilitates information coupling within layers while suppressing noise interference.

The hybrid architecture partially addresses the limitations of both Transformers and CNNs, combining local feature extraction with global context modelling capabilities. However, such architectures still face notable challenges. For example, in modelling deep interactions between dual-temporal features, they fail to fully capture the complex relationships and dynamic variations between dual-temporal image features, leading to inadequate representation of change regions. Additionally, during the dual-temporal feature extraction process in the backbone network, there is a lack of effective modelling of global context information. This limitation makes it difficult to fully integrate long-range dependencies and multi-scale features, thereby restricting the model’s accuracy and robustness in detecting complex change regions. Furthermore, as remote sensing image data volumes continue to grow, optimizing computational resources, reducing model complexity, and ensuring efficient operation for long-term, continuous monitoring tasks remain critical challenges. Although existing methods have improved computational efficiency to some extent, they still encounter significant computational costs when processing large-scale data.

We leverage the advantages of hybrid architectures combining CNN and Transformer and, based on the design concept of ChangeFormer, propose an improved change detection network—CGLCS-Net. This network reduces the number of parameters while enhancing the backbone network’s ability to model local and global features. Additionally, we introduce a dynamic interaction mechanism for dual-temporal feature modelling, further improving the detailed expression of change information. The main contributions of this paper are as follows:

(1) We propose the Global-Local Context-Aware Selector (GLCAS) module, which combines depth convolution with different receptive fields and an adaptive selection mechanism to capture both local details and global dependencies rather than merely modelling dense dependencies. GLCAS reduces computational complexity, significantly improving the model’s ability to extract multi-scale features in complex scenes and effectively addresses the challenge of insufficiently capturing change features at different scales in multi-temporal remote sensing images for change detection.
(2) We design the Subspace Self-Attention Fusion (SSAF) module, which dynamically models the differences between dual-temporal features to precisely focus on meaningful change regions in remote sensing images, addressing multi-view changes in remote sensing data. Guided by feature differences, SSAF enhances the model’s focus on change regions, improving its flexibility and accuracy when handling irregular boundaries and subtle changes.
(3) We performed comparisons with 10 models. We conducted ablation experiments on three primary change detection datasets, validating the robustness and efficiency of CGLCS-Net and achieving state-of-the-art performance results.

## 2. Methodology

**Figure 1:**
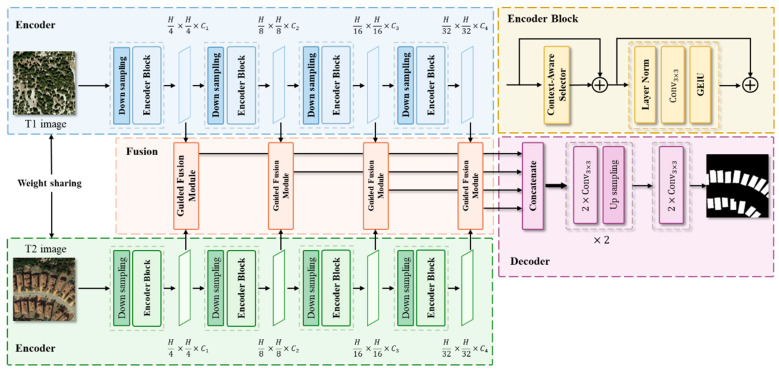
Overall Architecture of CGLCS-Net.

The overall architecture of CGLCS-Net comprises three parts: the Siamese Encoder, Feature Fusion module, and Decoder module, as shown in Figure 1.

### 2.1. Encoder

The encoder section utilizes a Siamese architecture with shared weights, significantly reducing the model’s parameter count and computational complexity compared to independent encoders while ensuring the consistent representation and context-awareness of dual-temporal features within the same semantic space. This provides a solid foundation for the precise modelling of differential features. This design focuses on the Global-Local Context-Aware Selector (GLCAS). The core of GLCAS is the combination of depthwise separable convolutions with different kernel sizes, allowing the network to comprehensively capture local details and global semantic information across different receptive fields at various stages. This design maintains low parameter count and computational complexity while meeting the extraction needs of multi-temporal and irregular change region multi-scale context features. The adaptive selection mechanism dynamically adjusts information fusion across different receptive fields, enhancing the model’s sensitivity to fine-grained changes.

### 2.2. Feature Fusion and Decoder

The feature fusion section employs the Subspace-based Self-Attention Fusion (SSAF) module, which focuses on dynamic interaction modelling between dual-temporal features. The SSAF module reduces computational complexity by decomposing dual-temporal features into different subspaces while using the difference features as query signals for the self-attention mechanism. This enables precise identification of key change regions within each subspace, removing noise generated by viewpoint and sensor variations and enhancing the network’s focus on meaningful change regions. The decoder section features a simple and efficient design, restoring spatial resolution through upsampling operations while concatenating and fusing features at various scales. Independent segmentation heads are used to produce the final change detection results, effectively ensuring the decoder remains computationally efficient.

The GLCAS and SSAF modules are described in detail as follows:

1. Global-Local Context-Aware Selector

**Figure 2:**
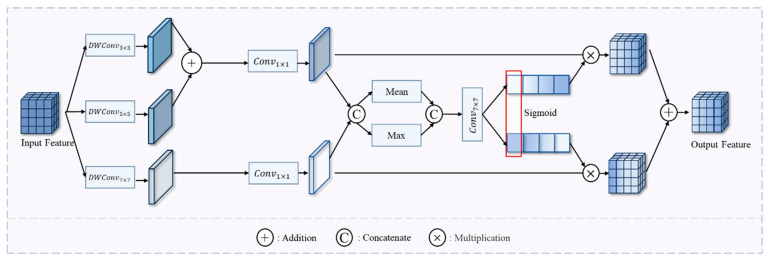
GLCAS (Global-Local Context-Aware Selector).

Building on Selective Kernel Networks [38], we replace the Transformer block in the ChangeFormer backbone to reduce the computational complexity and parameter count of feature extraction. We propose the Global-Local Context-Aware Selector (GLCAS), which introduces multi-scale depthwise separable convolutions to capture both local details and global context information. This approach dynamically adjusts the weight distribution of global and local features across different receptive fields in each feature extraction stage, as shown in Figure 2.

We designed parallel depthwise separable convolutions with scales of 3 × 3, 5 × 5, and 7 × 7 to extract multi-scale features, allowing the model to simultaneously focus on local details, medium-scale features, and global context information. The 7 × 7 convolution is combined with a dilation rate of 3 to enlarge the receptive field, as shown in Equations (1)–(3).

(1) $$small={DWConv}_{3\times 3}\left(x\right)$$

(2) $$medium={DWConv}_{5\times 5}\left(x\right)$$

(3) $$large={DWConv}_{7\times 7}^{dilation=3}\left(x\right)$$

where *DWConv* denotes the depthwise separable convolution, and *dilation* denotes the dilation rate.

An adaptive weighting strategy is employed in the feature fusion stage to dynamically modify the extracted multi-scale features. First, the small-scale and medium-scale features are summed and reduced in dimensionality via 1 × 1 convolutions, while the large-scale features undergo independent dimensionality reduction processing. This is shown in Equations (4) and (5):

(4) $${attn}_{1}={Conv}_{1\times 1}\left(small+medium\right)$$

(5) $${attn}_{2}={Conv}_{1\times 1}\left(large\right)$$

Secondly, by performing feature concatenation along the channel dimension, we combine global average pooling and max pooling operations for feature aggregation. The aggregated features are then processed through a 1 × 1 convolutional layer followed by a sigmoid activation function to generate adaptive weights, as formalized below:

(6) $$attn=Concat\left({attn}_{1}+{attn}_{2}\right)$$

(7) $$Avg_{attn}=Mean\left(attn\right)$$

(8) $$Max_{attn}=Max\left(attn\right)$$

(9) $$agg=Concat\left(Avg_{attn}+Max_{attn}\right)$$

(10) $$\sigma_{1},\sigma_{2}=f_{split}(Sigmoid\left({Conv}_{7\times 7}\left(agg\right)\right)$$

(11) $${Output}_{before}={Conv}_{1\times 1}\left(\sigma_{1}\cdot {attn}_{1}+\sigma_{2}\cdot {attn}_{2}\right)$$

where $f_{split}$ denotes the channel-wise split operation. The adaptive weights are parameterized by $\sigma_{1}$ and $\sigma_{2}$ through learnable transformations.

Finally, we incorporate DropPath [39] regularization after feature fusion to enhance model robustness and prevent overfitting. This mechanism randomly discards features along specific paths during training, thereby enforcing more substantial generalization capabilities. The formulation is given by:

(12) $$Output=DropPath\left({Output}_{before}\right)$$

2. Subspace-based Self-Attention Fusion Module

**Figure 3:**
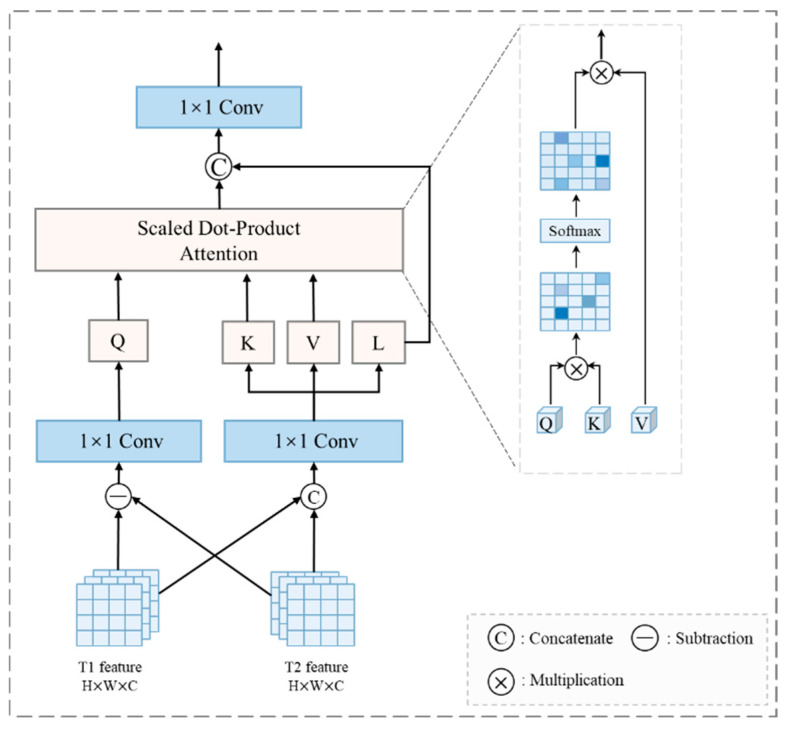
SSAF (Subspace-based Self-Attention Fusion module).

To establish dynamic spatiotemporal variation relationships between dual-temporal features and difference features, we propose a Subspace-based Self-attention Assimilation Fusion module (SSAF) as illustrated in Figure 3. The SSAF module synergistically integrates self-attention mechanisms with external query mechanisms through compact subspace design. Specifically, it projects input features into three subspaces, achieving computational complexity reduction.

In the feature decomposition stage, we sum the features from the T1 and T2 temporal phases, then project the aggregated features into three independent subspaces through 1 × 1 convolutional layers. The first subspace serves as the Key (*K*) to capture dynamic relationships between features, the second as the Value (*V*) to preserve salient change characteristics, while the third generates local residual features (denoted as *l*) to retain local details and original feature fidelity, as formalized in Equations (13) and (14):

(13) $$input_{feat}=Concat\left({feat}_{t1}+{feat}_{t2}\right)$$

(14) $$K,V,l={Conv}_{1\times 1}\left(input_{feat}\right)$$

During the feature subspace attention mechanism phase, the module explicitly models dynamic interactions between dual-temporal and difference features through attention computation between external query features *Q* and subspace features (Key *K* and Value *V*). This enables cross-feature-domain information fusion, where the different features are generated by taking the absolute value of the temporal feature differences. The mathematical formulation is given in Equation (15):

(15) $$Sub_{feat}=\left|{feat}_{t1}-{feat}_{t2}\right|$$

Building upon this framework, the query features *Q* are generated from the difference features through a 1 × 1 convolutional layer, which guides the attention mechanism to focus on change-sensitive regions. The formal implementation is given in Equation (16):

(16) $$Q={Conv}_{1\times 1}\left(Sub_{Feat}\right)$$

Subsequently, the attention weights are computed through dot products between query features *Q* and keys *K*, followed by Softmax normalization to ensure smoother weight distribution and improved optimization stability, as formalized in Equation (17). Finally, these weights are applied to values *V* to achieve aggregation and updating of dual-temporal change features:

(17) $$attn=Softmax\left(\frac{Q\cdot K^{T}}{\sqrt{d_{k}}}\right)\cdot V$$

where *Softmax* denotes the normalization function applied row-wise

Finally, the local residual features l and attention-enhanced global features attn are concatenated along the channel dimension, followed by a 1 × 1 convolutional layer for feature integration. The output features are subsequently fed into the decoder for change region extraction, as formalized in Equation (18):

(18) $$OutPut={Conv}_{1\times 1}\left(Concat\left(l,attn\right)\right)$$

## 3. Experiments

### 3.1. Dataset

We selected three widely applicable and representative remote sensing change detection datasets: LEVIR-CD [40], SYSU-CD [41], and S2Looking [42]. These datasets feature long temporal spans, multi-sensor sources, and multi-view characteristics. To facilitate fair comparisons with state-of-the-art methods, all remote sensing images were uniformly resized to 256 × 256 pixels, and standard dataset partitions were adopted to allocate training, validation, and test sets.

LEVIR-CD: Contains 637 high-resolution remote sensing images with original dimensions of 1024 × 1024 pixels, a spatial resolution of 0.5 m per pixel, and a temporal span of 5 to 14 years. The dataset primarily documents the growth and decline of buildings, covering diverse architectural types, including villa residences, high-rise apartments, small garages, and large warehouses. After resizing to 256 × 256 pixels, the dataset is partitioned into 7120 training images, 1024 validation images, and 2048 test images.

SYSU-CD: Contains 20,000 pairs of high-resolution remote sensing images captured in Hong Kong between 2007 and 2014, with original dimensions of 256 × 256 pixels and a spatial resolution of 0.5 m per pixel. The dataset records diverse change types, including new urban constructions, suburban expansion, pre-post-construction site changes, vegetation variations, road extensions, and marine development. It is partitioned into 12,000 training images, 4000 validation images, and 4000 test images.

S2Looking: Comprises 5000 dual-temporal satellite image pairs with off-nadir viewing angles from global rural areas. Original image dimensions are 1024 × 1024 pixels, with a spatial resolution of 0.5 to 0.8 m per pixel. The dataset focuses on land-cover changes in rural regions. After resizing, it is partitioned into 56,000 training images, 8000 validation images, and 16,000 test images.

### 3.2. Assessment Metric

The model’s accuracy is assessed on five standard evaluation metrics for change detection tasks [43]: Precision, Recall, F1 score, IoU (Intersection over Union), and OA (Overall Accuracy). These metrics are formulated as follows:

(19) $$\begin{array}{c} Precision=\frac{TP}{TP+FP} \end{array}$$

(20) $$\begin{array}{c} Recall=\frac{TP}{TP+FN} \end{array}$$

(21) $$\begin{array}{c} F1=\frac{2\times precision\times recall}{precision+recall} \end{array}$$

(22) $$\begin{array}{c} IoU=\frac{TP}{TP+FP+FN} \end{array}$$

(23) $$\begin{array}{c} OA=\frac{TP+TN}{TP+TN+FP+FN} \end{array}$$

where *TP* denotes the number of pixels in the change areas correctly extracted by the network, *TN* represents the number of pixels in the unchanged areas correctly extracted, *FP* indicates the number of unchanged area pixels incorrectly classified as change area pixels, and *FN* refers to the number of change area pixels incorrectly classified as unchanged area pixels.

### 3.3. Experimental Environment

CGLCS-Net is developed based on the PyTorch = 2.0.0 + cu117 framework and trained on an NVIDIA RTX 4070 GPU. In the experiments, the hyperparameters are configured as follows: the batch size is set to 8, the optimizer is Adamw, and the initial learning rate is set to 0.0001, which is dynamically adjusted using a multi-step decay strategy to enhance the flexibility of parameter optimization. To improve the model’s generalization capability, various data augmentation techniques are extensively applied, including random cropping, random rotation, horizontal flipping, Gaussian blur, random hue adjustment, random saturation adjustment, and random brightness adjustment.

To address the issue of extreme class imbalance in change detection tasks, a hybrid loss function [44] is designed to mitigate the impact of sample imbalance on model training. This hybrid loss function combines a weighted cross-entropy loss [45] and a Dice loss [46], and its specific definition is as follows:

(24) $$\begin{array}{c} L_{dice}=1-\frac{2\sum_{i=1}^{N}y_{i}{\hat{y}}_{i}}{\sum_{i=1}^{N}y_{i}+\sum_{i=1}^{N}{\hat{y}}_{i}} \end{array}$$

(25) $$\begin{array}{c} L_{wce}=-\frac{1}{N}\sum_{i=1}^{N}\sum_{c=1}^{C}w_{c}\left[y_{i}\cdot log\left({\hat{y}}_{i}\right)+\left(1-y_{i}\right)\cdot log\left(1-{\hat{y}}_{i}\right)\right] \end{array}$$

(26) $$\begin{array}{c} L_{total}={\alpha \times L}_{wce}+{\beta \times L}_{dice} \end{array}$$

where: $y_{i}$ denotes the true pixel value, $\hat{y}$ denotes the predicted pixel value, $w_{c}$ denotes the weight for class *C*, *α* and *β* are weight control parameters with values set to 1 and 0.5, and *N* denotes the total number of pixels.

### 3.4. Model Comparison

To thoroughly assess the performance of the CGLCS-Net model, it is compared with ten advanced change detection methods. These include CNN-based methods: FC-Siam-Conc, FC-Siam-Diff, DTCDSCN, SNUNet, DMINet, A2Net and ABMFNet [47], as well as Transformer-based methods: BIT, ChangeFormer, and EATDer.

The quantitative evaluation results show that CGLCS-Net achieved top-ranking scores compared to all ten advanced methods, as shown in Table 1, Table 2 and Table 3.

Table 1 presents the quantitative experimental results of the model on the LEVIR-CD dataset. Due to the long time span and the irregular shapes and scales of the change regions, this dataset places higher demands on the model’s ability to extract multi-scale features and model irregular regions. CGLCS-Net achieved the highest scores in Recall, F1, IoU, and OA across all evaluation metrics. In comparison to CNN-based models, FC-Siam-conc and FC-Siam-diff performed the poorest, suggesting that direct feature concatenation and difference methods are insufficient for feature capture. In contrast, DTCDSCN and ABMFNet, which incorporate spatiotemporal attention mechanisms, improved the model’s focus on change features, leading to better IoU and F1 scores. This indicates that spatiotemporal attention mechanisms can effectively help reduce misclassifications when dealing with long time spans and irregular regions. While A2Net achieved the highest Precision (92.96%), it had a relatively lower Recall (85.81%). On the other hand, CGLCS-Net demonstrated the optimal Recall value while maintaining 91.27% Precision through dynamic feature selection, confirming its advantage in feature extraction and processing using convolutional structures. When compared to Transformer-based models, CGLCS-Net also outperforms in comprehensive metrics, highlighting its superior ability to integrate global and local information. These results suggest that CGLCS-Net offers a significant advantage in extracting features from irregular regions with long time spans.

Table 2 presents the quantitative experimental results of various models on the SYSU-CD dataset. The SYSU-CD dataset is characterized by a wide variety of change types, a long time span, and a larger data volume. The experimental results show that CGLCS-Net achieved higher scores in Recall, F1, and IoU evaluation metrics. Compared to convolution-based models such as FC-Siam-conc, FC-Siam-diff, DTCDSCN, DMINet, A2Net, and ABMFNet, CGLCS-Net showed a slight decrease in Precision and OA (Overall Accuracy). Specifically, FC-Siam-conc and FC-Siam-diff achieved Precision scores of 83.51% and 86.14%, respectively, demonstrating strong precision but lower Recall. This suggests that these methods, which rely on difference calculation and feature concatenation, tend to overlook certain types of changes, failing to capture all changes comprehensively. In contrast, CGLCS-Net achieved 83.32% in Recall, a significant improvement, indicating that it can more comprehensively extract feature information from change regions, allowing it to identify more changes. Compared to Transformer-based models, CGLCS-Net also performed better in terms of balancing performance: while BIT (84.14%) and EATDer (81.02%) slightly outperformed CGLCS-Net in Precision, CGLCS-Net demonstrated a 7.88% and 0.97% improvement in F1 score over BIT (74.05%) and EATDer (80.96%), respectively, thanks to the effective noise reduction provided by its spatial-spectral attention mechanism (SSAF). This result confirms that the hybrid architecture of CGLCS-Net is better at coordinating global context modelling with local feature refinement, achieving a superior balance of performance in complex scenarios.

Table 3 presents the quantitative experimental results of the model on the S2Looking dataset. This dataset is characterized by varying ground-level viewpoints, complex rural object change patterns, and the absence of uniform boundaries. CGLCS-Net achieved the highest scores in Recall, F1, IoU, and OA across all evaluation metrics. While FC-Siam-conc achieved the highest Precision (84.23%), its Recall (34.18%) was significantly low, indicating that its strict difference threshold led to a high number of missed detections. FC-Siam-diff improved Recall to 50.62% by modifying the difference strategy, but its Precision dropped by 16.08%, reflecting the limited adaptability of simple feature operations in complex rural scenarios. In contrast, other convolution-based models such as DTCDSCN, SNUNet, DMINet, A2Net, and ABMFNet employed different strategies. Although these models exhibited lower Precision, they showed improvements in Recall and IoU, suggesting that the incorporation of attention mechanisms or enhanced multi-scale feature fusion can effectively improve the network’s ability to capture and locate features in scenes with varying viewpoints. When compared to Transformer-based models such as BIT, ChangeFormer, and EATDer, CGLCS-Net led in Recall (63.82%), F1 (64.35%), and IoU (47.43%), demonstrating its more comprehensive change detection capabilities. Although CGLCS-Net’s Precision (64.88%) is lower than that of ChangeFormer, its superior performance in F1 and IoU indicates that the model not only effectively captures detailed change features but also suppresses noise caused by viewpoint differences, thereby more accurately identifying change regions.

**Figure 4:**
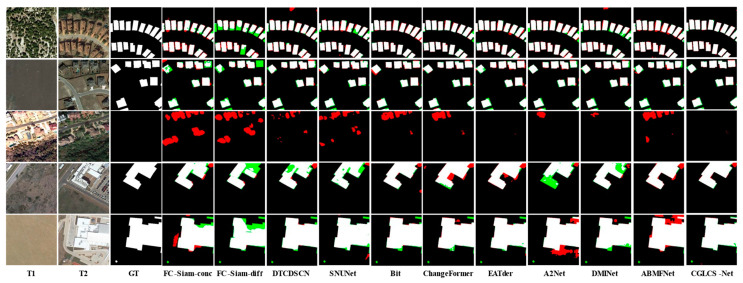
Visualization Results of LEVIR–CD. T1 and T2 represent images taken at different time points. GT denotes the ground truth labels, red indicates FP, green signifies FN, black represents TN, and white denotes TP.

Figure 4 presents the visualization results for the LEVIR-CD dataset. The first and second rows show scenes with small building clusters where building shadow interference is present. CGLCS-Net significantly reduces misclassification and omission when extracting edge details, leading to more precise segmentation results. The third row shows scenes with lighting changes, where all ten other models exhibit significant misclassification, while CGLCS-Net maintains accurate segmentation without any misclassification. The fourth and fifth rows represent large building scenes, where CGLCS-Net provides a more complete segmentation of building interiors.

**Figure 5:**
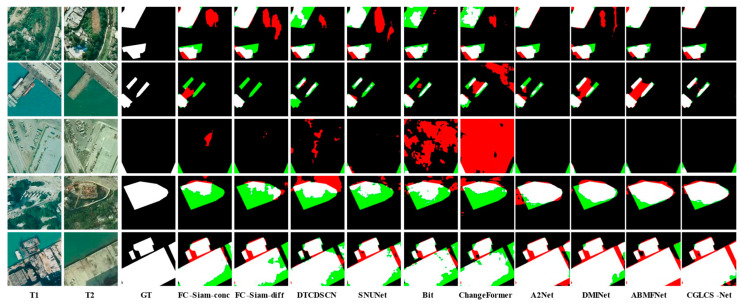
Visualization Results of SYSU-CD. T1 and T2 represent images taken at different time points. GT denotes the ground truth labels, red indicates FP, green signifies FN, black represents TN, and white denotes TP.

Figure 5 presents the visualization results for the SYSU-CD dataset. CGLCS-Net outperforms other comparison methods across various change types. The first and fourth rows show scenes with irregular buildings prone to tree occlusion and shadow interference. CGLCS-Net can capture the change regions more accurately, effectively reducing false positives and false negatives. The second, third, and fifth rows’ truth labels, red indicates FP, green signifies FN, black represents TN, and white denotes TP.

Show scenes from the port building areas. CGLCS-Net demonstrates more stable performance in overcoming interference from lighting variations, texture differences, and color discrepancies, significantly reducing misclassification and omission.

**Figure 6:**
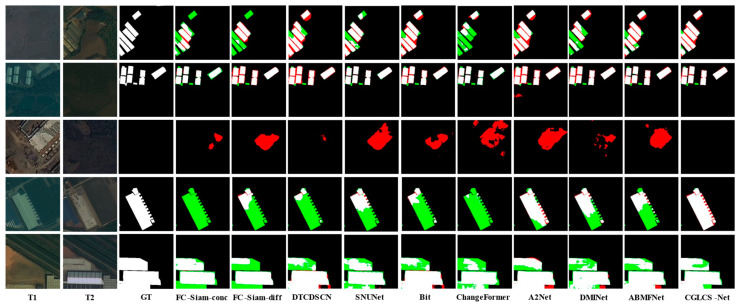
Visualization Results of S2Looking. T1 and T2 represent images taken at different time points. GT denotes the ground truth labels, red indicates FP, green signifies FN, black represents TN, and white denotes TP.

Figure 6 presents the visualization results for the S2Looking dataset. The first, second, and third rows show small building cluster scenes, where CGLCS-Net produces more complete detection results when facing different color variations. The fourth and fifth rows show large building scenes, where CGLCS-Net achieves the complete extraction of change regions despite the influence of viewpoint differences and boundary shadows.

### 3.5. Ablation Study

To thoroughly assess the effectiveness of the proposed modules and their contribution to the overall model performance, we conducted a series of ablation studies on the LEVIR-CD dataset, as shown in Table 4. We removed all Transformer Block modules from ChangeFormer and used them as the baseline (Baseline). In the feature fusion stage, we used only a simple concatenation strategy, significantly reducing the parameters and computational complexity but resulting in a 0.85% and 1.39% decrease in F1-score and IoU, respectively.

In the Baseline + GLCAS experiment, the parameter count increased from 10.71 M to 11.51 M, with F1-Score improving from 89.55% to 89.76% and IoU increasing from 81.09% to 81.42%. This demonstrates that the GLCAS module significantly enhances multi-scale feature representation through multi-scale depthwise separable convolutions and global-local dynamic fusion.

In the Baseline + SSAF experiment, the parameter count slightly increased to 11.28 M, with F1-Score rising to 89.91% and IoU increasing to 81.68%. This shows that the SSAF module effectively focuses on changing regions by modelling the dynamic interaction between dual-temporal and different features.

In the Baseline + GLCAS + SSAF experiment, the parameter count increased to 12.09 M, with F1-Score improving to 90.96% and IoU increasing to 83.43%. Compared to the Baseline model, IoU improved by 2.34%, proving that GLCAS and SSAF modules work synergistically in feature extraction and dynamic interaction modelling, significantly improving overall performance. Furthermore, compared to the original ChangeFormer model, CGLCS-Net (Baseline + GLCAS + SSAF) reduced the parameter count from 41.03 M to 12.09 M (a reduction of about 70.5%) while maintaining excellent detection performance. This demonstrates that CGLCS-Net excels not only in performance but also in lightweight design.

To further assess the impact of the pooling configuration in the Global-Local Context-Aware Selector (GLCAS), we conducted comparative experiments with different pooling methods (Max Pooling, Mean Pooling, and a combined pooling strategy), with the results shown in Table 5.

When Max Pooling was used to extract global context information, the F1-score was 89.79%, and the IoU was 81.47%, indicating that Max Pooling tends to preserve prominent features in the data and can effectively capture local extrema in the change regions. This demonstrates that Max Pooling outperforms the use of Mean Pooling alone. In contrast, Mean Pooling provides a smoother representation of global information, with F1-Score and IoU values of 89.54% and 81.06%, respectively. To combine the strengths of both methods, we used a combined pooling strategy, which resulted in a significant increase in F1-score to 90.96% and IoU to 83.43%. The combined pooling approach achieves complementarity between both methods, effectively capturing both significant and global smoothing features, significantly improving the GLCAS module’s ability to model complex change regions.

To further assess the impact of hyperparameters on model performance, we conducted multiple experiments on α and β in Equation (26). Table 6 presents the experimental results for the model under various combinations of these hyperparameters across several evaluation metrics. The results indicate that when α = 1 and β = 0.5, the model achieves the best accuracy, ensuring effective detection of changing regions while preventing an overemphasis on precision that could neglect recall. In contrast to other combinations of α and β, when the β weight is equal to or lower than α, the model exhibits lower recall and IoU but higher precision. This suggests that the model prioritizes pixel classification accuracy at the expense of its focus on changing regions.

### 3.6. Model Efficiency Analysis

We evaluated 11 models based on their parameter count (Params), floating-point operations (FLOPs), and inference time, as presented in Table 7. The image size used for the experiments was 3 × 256 × 256, with parameters measured in millions (M), floating-point operations in gigaflops (G), and inference time in seconds. CGLCS-Net, a model that combines convolutional and self-attention mechanisms, has 12.09 million parameters and 187.58 gigaflops of floating-point operations. In terms of computational efficiency, compared to the baseline model, ChangeFormer, CGLCS-Net reduces the parameter count by approximately 70%, floating-point operations by 7.5%, and inference time by 11.5%. Although CGLCS-Net’s computational complexity remains higher than most lightweight models, it demonstrates superior performance on the task. In the key evaluation metrics, the F1 score (90.96%) and Intersection over Union (IoU) (83.43%), CGLCS-Net outperforms all other models.

## 4. Conclusions

We propose CGLCS-Net, a high-resolution remote sensing image change detection network that integrates the Global-Local Context-Aware Selector (GLCAS) and the Subspace Self-Attention Fusion (SSAF) module. GLCAS addresses the challenge of balancing global context and local detail features in remote sensing images with complex scenes and varying-scale change features by combining multi-scale depthwise separable convolutions and an adaptive weighting mechanism. This significantly improves the model’s ability to extract multi-scale and irregular change regions. The SSAF module models the dynamic interaction between dual-temporal remote sensing image features and their differential features while preserving local residual features. It effectively handles the texture and spectral inconsistencies caused by sensor viewpoint differences and extensive periods, enhancing the detailed expression and precise modelling of change regions. The synergistic design of both modules effectively reduces the number of trainable parameters, significantly improving the model’s ability to analyze and detect complex change regions. Experimental results demonstrate that CGLCS-Net achieved optimal performance on the LEVIR-CD, SYSU-CD, and S2Looking public datasets, showcasing its superiority and robustness in handling diverse remote sensing change detection tasks. Future work will focus on optimizing the model structure and applying model compression techniques, including quantization and pruning, to reduce the floating-point operations of CGLCS-Net while preserving its existing performance. Furthermore, the feasibility of deploying CGLCS-Net for real-time, large-scale remote sensing image change detection on edge computing devices will be investigated.

## Figures and Tables

**Table 1:** Quantitative evaluation results on the LEVIR-CD dataset (UNIT: %).

Methods	LEVIR-CD				
	Precision	Recall	F1	IoU	OA
FC-Siam-conc	84.22	88.11	86.12	75.63	98.55
FC-Siam-diff	86.36	84.11	85.22	74.25	98.51
DTCDSCN	90.18	88.94	89.56	81.09	98.94
SNUNet	91.31	88.67	89.97	81.77	98.99
BIT	89.24	89.37	89.31	80.68	98.92
ChangeFormer	92.05	88.80	90.40	82.48	99.04
EATDer	91.58	88.74	90.14	82.05	99.01
A2Net	92.96	85.81	89.24	80.58	98.95
DMINet	90.85	88.96	89.90	81.64	98.98
ABMFNet	90.15	88.97	89.55	81.08	98.94
**CGLCS** **-Net**	91.27	**90.65**	**90.96**	**83.43**	**99.08**

**Table 2:** Quantitative evaluation results on the SYSU-CD dataset (UNIT: %).

Methods	SYSU-CD				
	Precision	Recall	F1	IoU	OA
FC-Siam-conc	83.51	74.09	78.52	64.64	90.44
FC-Siam-diff	86.14	67.44	75.66	60.85	89.76
DTCDSCN	81.99	68.24	74.49	59.35	89.97
SNUNet	77.32	80.11	78.69	64.87	89.77
BIT	84.14	66.12	74.05	58.79	89.07
ChangeFormer	79.37	71.33	75.13	60.17	88.86
EATDer	81.02	80.90	80.96	68.01	91.03
A2Net	84.69	78.75	81.63	68.93	91.63
DMINet	84.19	78.10	81.19	68.11	91.37
ABMFNet	81.15	80.29	80.71	67.66	89.77
**CGLCS-Net**	80.59	**83.32**	**81.93**	**69.40**	91.33

**Table 3:** Quantitative evaluation results on the S2LOOKING dataset (UNIT: %).

Methods	S2Looking				
	Precision	Recall	F1	IoU	OA
FC-Siam-conc	84.23	34.18	48.63	32.13	99.12
FC-Siam-diff	68.15	50.62	58.09	40.93	99.11
DTCDSCN	61.27	57.70	59.43	42.28	99.04
SNUNet	67.51	50.68	57.89	40.74	99.10
BIT	63.24	59.19	61.53	44.44	99.09
ChangeFormer	69.88	40.21	51.05	34.27	99.06
EATDer	54.88	62.05	58.24	41.09	98.92
A2Net	59.67	57.75	58.70	41.54	99.01
DMINet	58.01	59.28	58.63	41.48	98.98
ABMFNet	57.64	61.25	61.36	44.33	98.81
**CGLCS-Net**	64.88	**63.82**	**64.35**	**47.43**	**99.14**

**Table 4:** Ablation study results of the modules.

Methods	Params (M)	F1 (%)	IoU (%)
ChangeFormer	41.03	90.40	82.48
Baseline	10.71	89.55	81.09
Baseline + GLCAS	11.51	89.76	81.42
Baseline + SSAF	11.28	89.91	81.68
Baseline + GLCAS + SSAF	12.09	90.96	83.43

**Table 5:** Ablation study results of GLCAS Pooling configuration (UNIT: %).

GLCAS	F1	IoU
Max	89.79	81.47
Mean	89.54	81.06
Max + Mean	90.96	83.43

**Table 6:** Quantitative Results of the Loss Function Ablation Study (Unit: %).

α	β	Precision	Recall	F1	IoU	OA
1	1	90.35	87.87	89.09	80.33	98.90
1	0.5	91.27	90.65	90.96	83.43	99.08
0.5	0.5	90.66	87.64	89.12	80.38	98.91
0.5	1	90.01	87.06	88.51	79.39	98.84

**Table 7:** Quantitative Evaluation Results of Model Efficiency.

Methods	Params (M)	Flops (G)	Times (S)	F1 (%)	IoU (%)
FC-Siam-conc	1.55	5.33	0.0085	86.12	75.63
FC-Siam-diff	1.35	4.73	0.0074	85.22	74.25
DTCDSCN	31.25	13.22	0.0136	89.56	81.09
SNUNet	12.03	54.83	0.0152	89.97	81.77
BIT	3.496	10.63	0.0131	89.31	80.68
EATDer	6.06	23.45	0.0320	90.14	82.05
A2Net	3.048	3.781	0.0210	89.24	80.58
DMINet	6.24	14.55	0.0201	89.90	81.64
ChangeFormer	41.03	202.78	0.0347	90.40	82.48
ABMFNet	47.13	66.17	0.0223	89.55	81.08
CGLCS-Net	12.09	187.58	0.0307	90.96	83.43

## Data Availability

Data are contained within the article.
